# Cardiac extracellular volume fraction in cats with preclinical hypertrophic cardiomyopathy

**DOI:** 10.1111/jvim.16067

**Published:** 2021-02-26

**Authors:** Ryan C. Fries, Saki Kadotani, Stephanie C. J. Keating, Jonathan P. Stack

**Affiliations:** ^1^ Department of Veterinary Clinical Medicine University of Illinois Urbana‐Champaign Illinois USA

**Keywords:** cardiac fibrosis, feline, magnetic resonance imaging, strain, T1 mapping

## Abstract

**Background:**

Cardiac magnetic resonance imaging (CMR) allows for detection of fibrosis in hypertrophic cardiomyopathy (HCM) by quantification of the extracellular volume fraction (ECV).

**Hypothesis/Objectives:**

To quantify native T1 mapping and ECV in cats. We hypothesize that native T1 mapping and ECV will be significantly increased in HCM cats compared with healthy cats.

**Animals:**

Seventeen healthy and 12 preclinical HCM, age‐matched, client‐owned cats.

**Methods:**

Prospective observational study. Tests performed included indirect blood pressure, CBC, biochemical analysis including total thyroid, urinalysis, transthoracic echocardiogram, and CMR. Cats were considered healthy if all tests were within normal limits and a diagnosis of HCM was determined by the presence of left ventricular concentric hypertrophy ≥6 mm on echocardiography.

**Results:**

There were statistically significant differences in LV mass (healthy = 5.87 g, HCM = 10.3 g, *P* < .0001), native T1 mapping (healthy = 1122 ms, HCM = 1209 ms, *P* = .004), and ECV (healthy = 26.0%, HCM = 32.6%, *P* < .0001). Variables of diastolic function including deceleration time of early diastolic transmitral flow (DTE), ratio between peak velocity of early diastolic transmitral flow and peak velocity of late diastolic transmitral flow (E : A), and peak velocity of late diastolic transmitral flow (A wave) were significantly correlated with ECV (DTE; *r* = 0.73 *P* = .007, E : A; *r* = −0.75 *P* = .004, A wave; *r* = 0.76 P = .004).

**Conclusions and Clinical Importance:**

Quantitative assessment of cardiac ECV is feasible and can provide additional information not available using echocardiography.

**Abbreviations**
A: peak velocity of late diastolic transmitral flow
CMR: cardiac magnetic resonance imaging
DTE: deceleration time of early diastolic transmitral flow
E : A: ratio between E and A
ECV: extracellular volume
E/SrCE: ratio between peak velocity of early diastolic transmitral flow and peak circumferential strain rate during early diastole
E/SrRE: ratio between peak velocity of early diastolic transmitral flow and peak radial strain rate during early diastole
GLPS: global longitudinal strain
IVSd: interventricular septal thickness in diastole
IVSs: interventricular septal thickness in systole
LAA: left auricular appendage
LA : Ao: ratio between LA_SAX_ and Ao
LA FS: left atrial fractional shortening
LVFWd: left ventricular free wall thickness in diastole
LVIDd: left ventricular internal dimension in diastole
SrCE: peak circumferential strain rate during early diastole
SrRE: peak radial strain rate during early diastole

## 1 INTRODUCTION

Hypertrophic cardiomyopathy (HCM) is the most common heart disease in cats.^1^, ^2^ It is characterized by variable patterns and distributions of left ventricular (LV) hypertrophy.^3^ Hallmark histopathologic lesions include myofiber disarray, small coronary arteriosclerosis, and interstitial and replacement fibrosis.^4^ In humans with HCM, myocardial interstitial changes can be focal or global and the final common end point of irreversible fibrosis has been linked to increased risk of cardiac complications.^5^, ^6^, ^7^ Cardiac magnetic resonance imaging (CMR) can be used to noninvasively assess myocardial fibrosis using late gadolinium enhancement (LGE); however, quantitative evaluation is limited.^8^, ^9^, ^10^, ^11^ Although LGE has been performed in normal cats and Maine Coon cats with mild to severe HCM, only 1 cat displayed focal LGE and there was no difference in overall myocardial contrast enhancement between normal cats and cats with HCM.^12^ This study indicates that LGE can detect myocardial fibrosis but is only useful for detection of focal fibrosis, where a discrete area of fibrosis is surrounded by normal myocardium. As such, in order to detect fibrosis using LGE, normal myocardium is necessary to establish a standard of reference. In cases where the pattern of fibrosis is diffuse and limited or no normal myocardium is available for reference, LGE cannot be utilized.

One method to overcome the limitation of LGE to detect diffuse fibrosis is quantitative myocardial mapping. T1 mapping measures the longitudinal or spin‐lattice relaxation time, which is determined by how rapidly protons reequilibrate their spins after an excitation radiofrequency pulse. All tissues have inherent T1 relaxation times that are based on a composite of their cellular and interstitial components.^8^ The 2 most important biological determinants of an increase in native T1 values are interstitial edema secondary to infarction with associated cellular destruction and increased interstitial space from fibrosis.^13^ Native T1 values are a composite signal of myocytes and extracellular volume (ECV), whereas contrast‐enhanced T1 mapping can specifically calculate the ECV fraction. Gadolinium‐based contrast agents are distributed throughout the extracellular space and shorten T1 relaxation times of the myocardium proportional to the local concentration for gadolinium.^14^ Areas of fibrosis and scar will therefore exhibit shorter T1 relaxation times, after contrast administration.^15^ Quantification of the ECV can be determined according to the formula:

$$\mathrm{ECV}=\left(1-\text{hematocrit}\right)\;\frac{\frac{1}{\text{post contrast}\;T1\;\text{myocardium}}-\frac{1}{\;\text{native}\;T1\;\text{myocardium}}}{\frac{1}{\text{post contrast}\;T1\;\text{blood}}-\frac{1}{\text{native}\;T1\;\text{blood}}}.$$

An increased ECV is due to excessive collagen deposition and is highly correlated with histological measures of collagen and fibrosis.^16^, ^17^, ^18^

The use of CMR to evaluate HCM in people has determined that native T1 values are prolonged and ECV increased in HCM.^19^, ^20^ T1 mapping and ECV assessment have the advantage of assessing fibrosis noninvasively and can detect diffuse fibrosis more accurately than LGE. The objective of this study was to quantify myocardial fibrosis, namely T1 mapping and ECV, in healthy and preclinical HCM cats using CMR and their association with echocardiographic variables.

## 2 MATERIALS AND METHODS

The study protocol was reviewed and approved by the Institutional Animal Care and Use Committee (Protocol #17281) at the University of Illinois at Urban‐Champaign.

### 2.1 Cats, clinical examinations, and group assignment

Twenty‐nine client‐owned cats were prospectively studied and recruited over a 2‐year period and emphasis was placed on recruiting older normal cats. Tests performed included physical examination, indirect blood pressure by Doppler method, CBC, biochemical analysis including total thyroid, urinalysis, transthoracic echocardiogram, and CMR with contrast. Cats were considered healthy if all diagnostic tests were within normal limits and a diagnosis of preclinical HCM was determined by the presence of either focal or generalized LV concentric hypertrophy ≥6 mm on echocardiography.^21^ All cats were preclinical and asymptomatic at the time of evaluation. None of the healthy cats or preclinical HCM cats were receiving medications other than topical heartworm and flea prevention.

### 2.2 Echocardiography

All transthoracic echocardiographic examinations were performed by a single investigator (RCF), using a digital ultrasound systemic (Vivid E95, GE Medical Systems, Waukesha, Wisconsin), equipped with a 12‐MHz phased‐array transducer with simultaneous ECG monitoring. Awake, unsedated cats were positioned in right, and then left lateral recumbency on a raised table with a central opening. All images and cine loops were digitally stored and transferred to a separate workstation (EchoPAC BT13 version 113.1.3 software, GE Medical Systems) for off‐line analysis. Each study was analyzed by the same observer (R.C. Fries) at the end of the recruitment period in random order. All studies were labeled by random identification number only and each measurement was repeated 5 times and the mean values were used for statistical analysis. Echocardiographic studies were performed before CMR on the same day.

Assessment of LV size and function was performed using standard right parasternal short‐axis and long‐axis views, and left apical parasternal long‐axis views.^22^ Two‐dimensional variables measured included LV internal dimensions at end‐diastole (LVIDd) and end‐systole (LVIDs), LV free‐wall thickness at end‐diastole (LVFWd) and end‐systole (LVFWs) and interventricular septal thickness at end‐diastole (IVSd) and end‐systole (IVSs). The LV fractional shortening was calculated using the following formula: LV − FS = [LVIDd − LVIDs]/LVIDd × 100%.

Assessment of left atrial (LA) size was performed from standard right parasternal long‐axis and short‐axis views. Variables measured included LA diameter (LA_SAX_) and aortic diameter (Ao) measured from a right parasternal short‐axis view in early diastole timed to the earliest frame in which the closed aortic value cusps could be visualized. The ratio between LA_SAX_ to Ao (LA : Ao) was calculated. Additionally, the LA septal‐to‐free wall dimension maximum (LAD_Max_) and minimum (LAD_Min_) were measured from the right parasternal long‐axis 4‐chamber view. The LAD_Max_ and LAD_Min_ were measured mid‐chamber approximately parallel to the mitral annuls at end LV systole immediately before mitral valve opening (LAD_Max_) and end LV diastole immediately after mitral valve closure (LAD_Min_). The LA fractional shortening was calculated using the following formula: LA FS = [LAD_Max_ − LAD_Min_]/LAD_Max_ × 100%. Left atrial appendage velocities, emptying (LAA emptying) and filling (LAA filling), were recorded using pulsed‐wave Doppler from the left cranial short‐axis view with the sampling gate placed as proximal the entrance of the LAA as possible.

Transmitral velocities were recorded using pulsed‐wave Doppler from a left apical parasternal long‐axis view with the sampling gate place in line with color Doppler flow at the level of the open mitral valve tips. Variables measured included isovolumic relaxation time (IVRT), peak velocity of early diastolic transmitral flow (E), deceleration time of early diastolic transmitral flow (DTE), and peak velocity of late diastolic transmitral flow (A). Ratio between peak E to peak A (E : A) was calculated.

Tissue Doppler imaging was performed with the highest available transducer frequency to record the velocity of lateral mitral annular motion from the left apical parasternal long‐axis view with the sampling gate placed on the lateral mitral annulus. The following variables were measured: peak early diastolic velocity (E′), peak late diastolic velocity (A′), and peak systolic velocity (S′).

Speckle tracking postprocessing was used to evaluate circumferential, radial, and global longitudinal strain. The peak systolic radial and circumferential strains (SR and SC), and the peak radial and circumferential strain rates during the systole and early diastole (SrRs, SrRE, SrCs, and SrCE) were measured at the level of papillary muscles. The variables E/SrCE and E/SrRE were derived from the E wave divided by the SrRE and SrCE, respectively. Global longitudinal strain was measured using the left apical 4‐chamber, left apical 2‐chamber, and left apical 5‐chamber views. All strain and strain rate variables were calculated by 6 segmental values (anteroseptum, anterior, lateral, posterior, inferior, and septum). A global value was calculated as the mean value in all 6 segments and an overall global longitudinal strain was calculated as the average of the global longitudinal strain values from the 3 long‐axis apical views.

### 2.3 Cardiac magnetic resonance imaging

The morning of the CMR procedure, all cats had 1 to 2 mL of blood drawn for determination of hematocrit, before being placed under general anesthesia. Premedication protocols were determined by a board‐certified anesthesiologist (S. Kadotani) based on clinical assessment and cardiovascular status. Anesthesia was induced with alfaxalone 2 mg/kg IV to effect and maintained with isoflurane in 100% oxygen following intubation. Cats were monitored throughout the CMR using ECG, pulse oximetry, end‐tidal carbon dioxide, direct arterial blood pressure monitoring, and assessment of anesthetic depth, heart rate, respiratory rate, and temperature with values recorded every 5 minutes. Lactated Ringer's solution was administered at 5 mL/kg/hour during the anesthetic period in healthy cats and 2.5 mL/kg/hour in HCM affected cats.

All cats underwent CMR studies performed on a MAGNETOM Skyra 3T scanner with a 4‐channel phased array flex coil (software version syngo MR E11 Siemens Healthcare, Erlangen, Germany). Scout images were used to identify the long‐ and short‐axis views of the left ventricle as well as the 2‐ and 4‐chamber views of the heart. Thereafter, cine‐images of 3 long‐axis views (4‐chamber, 2‐chamber, and 3‐chamber view) were acquired using a balanced steady‐state precession sequence in combination with parallel imaging and retrospective gating during an expiratory breath‐hold. Frequency scout scans were performed before each cine image and optimal frequency was subjectively assessed and determined by the operator (RCF). Frequencies (−200 Hz to +200 Hz) were optimally adjusted before each cine loop based on the frequency scout.^23^ Left ventricular systolic function and morphology imaging was accomplished utilizing with whole‐heart coverage (aortic root to left ventricular apex) of gapless short‐axis slices, with retrospective gating and multiple end‐expiratory breath holds. T1 mapping was performed from a short‐axis plane at the base and mid‐left ventricular chamber using a single breath hold, ECG‐triggered, modified Look‐locker inversion recovery sequence. The T1 maps were acquired before and 15‐minutes after contrast injection of 0.2 mL/kg (0.1 mmol/kg) gadopentetate dimeglumine (Magnevist; Bayer Healthcare, Wane, New Jersey). Late gadolinium enhancement images were obtained 10‐minutes after the bolus of gadopentetate dimeglumine using an inversion‐recovery gradient echo technique. Inversion time was individually determined based on TI prep pulse sequencing before contrast administration and manually adjusted (260‐480 ms) to null the myocardium and enhance any areas of contrast uptake.

**FIGURE 1:**
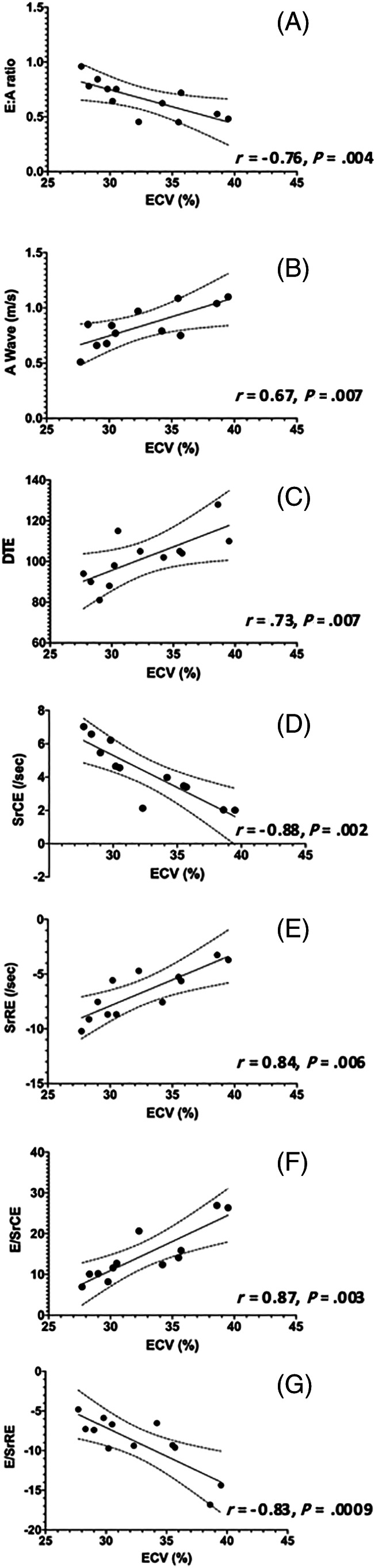
Representative images of mid‐ventricular native T1 mapping in 2 patients with high versus low native T1 values. Evaluation of native T1 times was determined from 4 manually drawn regions of interests as shown. For the hypertrophic cardiomyopathy (HCM) patient, native T1 times = 1240 ms, extracellular volume fraction (ECV) = 32.3%, and left ventricular mass = 15.3 g. For the normal patient, native T1 time = 1132 ms, ECV = 23.4%, and left ventricular mass = 7.5 kg

Left ventricular volume, functional analysis, and mass calculation were performed using commercially available software, (Argus, Siemens Healthcare). Manual segmentation of the epicardial and endocardial borders of the LV in the short axis were outlined from the CMR derived images. Ejection fraction, end‐diastolic volume, end‐systolic volume, and mass were calculated by manually tracing the epicardial and endocardial contours of the LV in each phase from end‐diastole to end‐systole. End‐diastole was determined by visually selecting the phase with the largest LV volume and end‐systole was determined by visually selecting the phase with the smallest LV volume. Papillary muscles were included in the LV cavity and thus not counted towards LV mass. Segmental myocardial wall thickness was manually measured in end‐diastole on basal and mid‐chamber images. Left ventricular segments with LGE were qualitatively evaluated and enhancement was defined as 6 standard deviations above the manually selected normal area (maximally suppressed myocardium on TI scout). Native and postcontrast MOLLI images were processed using commercially available software (cvi^42^, version 5.10, Circle Cardiovascular Imaging Inc, Calgary, Alberta, Canada). Four, manually drawn, regions of interest were used to create individual regional T1 values and ECV in both the basal and mid‐ventricular slice. The septum was divided into 2 regions, as was the free wall including the papillary muscles (Figure 1). Global T1 and ECV values were calculated as the average all regions in both slices.

### 2.4 Statistical analysis

Statistical analyses were performed using commercially available software (GaphPad Prism, version 8, GraphPad Software Inc, San Diego, California; SAS version 9.4, Cary, North Carolina). Continuous variables were expressed as mean ± SD when normally distributed, or median and range when the distribution was non‐normal. Normality was assessed using the Shapiro‐Wilk test. Groups were compared using unpaired Student's *t* test or Mann‐Whitney test. Within each group, Pearson's correlation and bivariate linear regression were used to evaluate the relationship between echocardiographic variables, age, weight, native T1 time, ECV, and left ventricular mass. Multiple linear regression was used to evaluate the relationship between diastolic variables, ECV, and age. Repeated measures 1‐way ANOVA, of the manually drawn regions of interest, was used to evaluate for differences in the distribution of ECV. Inter‐ and intraobserver variability was determined by having all echocardiographic and CMR studies measured twice, 10 months apart by 1 investigator (R.C. Fries) and once by another investigator (J.P. Stack) each blinded to the other's results. Intra‐ and interobserver variability was determined by the coefficient of variation utilizing the root means squared method ([coefficient of variation] = 100 × $\sqrt{{\frac{\sum \left(d/m\right)}{2n}}^{2}}$), where *d* is the difference between 2 paired measurements and *m* is the mean of the paired measurements. Statistical significance was defined for *P* values <.05. Receiver operating characteristic (ROC) analysis was performed to assess the diagnostic accuracy of native T1 time, ECV, and left ventricular mass to detect the presence of HCM. Diagnostic cutoffs for each variable were chosen on the basis of the highest of various combinations of sensitivity and specificity using Youden's index (Y = sensitivity + specificity − 1).

## 3 RESULTS

Demographic data and results of physical examination are summarized in Table 1. The study sample was comprised of 17 healthy cats and 12 cats with HCM. There were no differences between groups related to complete blood count, biochemistry profile, total thyroid, urinalysis, or demographic data, except for presence of a heart murmur detected more frequently in HCM cats. Two HCM cats had single premature ventricular complexes, which did not require treatment, noted during screening echocardiograms. None of the HCM cats or healthy cats had ectopic heart rhythms during anesthesia for their CMR studies. Comparison of echocardiographic variables is summarized in Table 2. Complete 2‐dimensional and color Doppler echocardiographic studies were successfully obtained in all cats. Two repeated measures, instead of 5, for E and A waves were obtained in 1 healthy cat. The remaining 16 healthy cats and all HCM cats had 5 repeated measures for all variables, including all diastolic and strain variables. Comparison of CRM variables is summarized in Table 3. Complete CMR studies were acquired in all cats. No cats had evidence of LGE. The median time required to complete the CRM studies was 40 minutes (30‐47 minutes). There were significant differences in left ventricular mass, native T1 time, postcontrast T1 time, and ECV between groups. No significant differences in the distribution of ECV were found. Inter‐ and intraobserver measurement variability for echocardiographic and CMR variables are summarized in Table 4.

**TABLE 1:** Demographic variables in 17 healthy and 12 cats with hypertrophic cardiomyopathy (HCM)

Variable	Healthy	HCM	*P*
Age (year)	6.0 ± 3.5	7.9 ± 4.9	.26
Body weight (kg)	5.0 ± 1.0	5.8 ± 1.2	.55
Sex (female : male)	7 : 10	5 : 7	.99
Heart rate (/min)	190 (183‐197)	184 (172‐187)	.27
Systolic heart murmur	0 (0%)	9 (75%)	.03
Doppler blood pressure (mm Hg)	135 ± 14	140 ± 27	.52

*Note*: Mean ± SD or median (5th and 95th percentiles) for continuous data and number or percentage for frequency data.

**TABLE 2:** Echocardiographic variables in 17 healthy and 12 cats with hypertrophic cardiomyopathy (HCM)

Variable	Healthy	HCM	*P*
LVIDd (mm)	15.19 ± 1.18	14.56 ± 1.74	.29
LVIDs (mm)	7.58 ± 1.10	7.84 ± 1.17	.55
LV FS (%)	50.2 ± 5.5	48.2 ± 5.1	.005
IVSd (mm)	4.31 ± 0.40	6.55 ± 0.87	<.0001
IVSs (mm)	6.52 (6.15‐6.86)	7.32 (6.09‐8.74)	.11
LVFWd (mm)	4.30 ± 0.53	7.49 ± 0.43	<.0001
LVFWs (mm)	6.81 ± 0.80	9.65 ± 1.46	<.0001
LAD_Max_ (mm)	13.62 ± 1.03	16.40 ± 2.07	.001
LAD_Min_ (mm)	10.51 ± 0.84	13.57 ± 1.75	<.0001
LA FS (%)	22.8 ± 3.0	17.3 ± 1.9	.0002
LA_SAX_ (mm)	12.24 ± 1.20	14.72 ± 1.80	.001
Ao (mm)	9.57 ± 1.04	10.55 ± 0.57	.002
LA : Ao	1.25 ± 0.06	1.43 ± 0.10	<.0001
LAA emptying (m/s)	0.52 ± 0.13	0.37 ± 0.08	.01
LAA filling (m/s)	0.47 ± 0.09	0.37 ± 0.07	.02
E (m/s)	0.64 ± 0.10	0.53 ± 0.06	.003
A (m/s)	0.60 ± 0.16	0.84 ± 0.18	.002
E : A	1.10 ± 0.19	0.67 ± 0.16	<.0001
DTE (m/s)	86.5 ± 18.8	101.7 ± 12.8	.02
DTE slope (m/s^2^)	7.69 ± 1.85	5.89 ± 1.96	.05
E′ (m/s)	0.07 ± 0.02	0.05 ± 0.02	.004
A′ (m/s)	0.06 ± 0.02	0.07 ± 0.02	.07
AV max (m/s)	0.87 ± 0.16	1.25 ± 0.54	.02
PV max (m/s)	0.85 ± 0.13	1.04 ± 0.29	.03
GLPS_LAX (%)	−18.9 ± 3.9	−14.0 ± 5.1	.01
GLPS_4CH (%)	−20.7 ± 3.5	−14.1 ± 6.8	.0003
GLPS_2CH (%)	−20.1 (−22.6 to −19.2)	−18.0 (−19.1 to −10.9)	.001
GLPS_AVE (%)	−19.6 ± 2.8	−15.6 ± 5.1	.004
SC (%)	−21.6 ± 4.75	−16.5 ± 3.90	.006
SrCs (/s)	−4.75 ± 1.13	−5.64 ± 1.71	.12
SrCE (/s)	5.83 ± 1.11	4.29 ± 1.77	.01
SR (%)	56.1 ± 22.6	44.9 ± 30.2	.21
SrRs (/s)	6.65 (5.84‐9.48)	6.85 (4.53‐9.13)	.51
SrRE (/s)	9.05 ± 3.04	−6.65 ± 2.28	.04
E/SrCE	10.39 ± 1.52	14.68 ± 6.64	.02
E/SrRE	−6.28 ± 1.02	−8.98 ± 3.51	.009

*Note*: Data are expressed as mean ± SD or median (5th and 95th percentiles).

Abbreviations: A, peak velocity of late diastolic transmitral flow; A′, peak late diastolic velocity of lateral mitral annulus; Ao, aortic short‐axis dimension in diastole; AV max, peak aortic flow velocity; DTE, deceleration time of early diastolic transmitral flow; DTE Slope, slope of DTE; E, peak velocity of early diastolic transmitral flow; E′, peak early diastolic velocity of lateral mitral annulus; E : A, ratio between E and A; E/SrCE, ratio between E and SrCE; E/SrRE, ratio between E and SrRE; GLPS_AVE, overall average of global longitudinal strain obtained from 3 apical long‐axis views; GLPS_2CH, global longitudinal strain from apical 2‐chamber view; GLPS_4CH, global longitudinal strain from aplical 4‐chamber view; GLPS_LAX, global longitudinal strain from apical long‐axis view; IVSd, interventricular septal thickness in diastole; IVSs, interventricular septal thickness in systole; LAD_Max_, maximum left atrial septal‐to‐free wall dimension in right parasternal long‐axis 4‐chamber view; LA_Min_, minimum left atrial septal‐to‐free wall dimension in right parasternal long‐axis 4‐chamber view; LA_SAX_, left atrium short‐axis diameter in diastole; LAA emptying, peak velocity blood flow leaving the left auricular appendage during atrial contraction; LAA filling, peak velocity of blood flow entering the left auricular appendage after atrial contraction; LA : Ao, ratio between LA_SAX_ and Ao; LA FS, left atrial fractional shortening; LV FS, left ventricular fractional shortening; LVFWd, left ventricular free wall thickness in diastole; LVFWs, left ventricular free wall thickness in systole; LVIDd, left ventricular internal dimension in diastole; LVIDs, left ventricular internal dimension in systole; PV max, peak pulmonic flow velocity; SrCE, peak circumferential strain rate during early diastole; SrRE, peak radial strain rate during early diastole.

**TABLE 3:** Cardiac magnetic resonance variables in 17 healthy and 12 cats with hypertrophic cardiomyopathy (HCM)

Variable	Healthy	HCM	*P*
LVEDV (mL)	5.50 ± 1.15	5.92 ± 1.19	.34
LVESV (mL)	2.91 ± 0.71	3.43 ± 0.67	.06
LV SV (mL)	2.61 ± 0.75	2.48 ± 0.82	.65
LV EF (%)	47.2 ± 8.1	41.3 ± 8.8	.07
LV mass (g)	5.87 ± 1.15	10.34 ± 1.97	.0004
LV native T1 time (ms)	1122 ± 30.4	1209 ± 61.1	<.0001
LV post T1 time (ms)	596 ± 25.4	568 ± 44.5	.04
LV ECV (%)	26.0 ± 2.0	32.6 ± 4.0	<.0001
HCT (%)	42 ± 3	41 ± 5	.39
HR (bpm)	110 ± 8	112 ± 9	.6

*Note*: Data are expressed as mean ± SD.

Abbreviations: HCT, hematocrit; HR, heart rate; LV ECV, left ventricular extracellular volume fraction; LVEDV, left ventricular end‐diastolic volume; LV EF, left ventricular ejection fraction; LVESV, left ventricular end‐systolic volume; LV SV, left ventricular stroke volume.

**TABLE 4:** Intra‐ and interobserver variability measurements for all echocardiographic and Cardiac magnetic resonance imaging (CMR) variables in healthy and hypertrophic cardiomyopathy (HCM) affected cats

Variable	Intraobserver CV%	Interobserver CV%
LVIDd (mm)	3.56	4.21
LVIDs (mm)	5.31	6.51
LV FS (%)	5.67	6.21
IVSd (mm)	4.22	5.13
IVSs (mm)	5.19	6.62
LVFWd (mm)	3.55	3.67
LVFWs (mm)	5.52	6.89
LAD_Max_ (mm)	4.13	4.86
LAD_Min_ (mm)	5.78	7.12
LA FS (%)	5.33	6.71
LA_SAX_ (mm)	4.42	4.69
Ao (mm)	1.63	1.79
LA : Ao	4.04	4.72
LAA emptying (m/s)	0.88	1.01
LAA filling (m/s)	1.01	1.26
E (m/s)	3.23	3.45
A (m/s)	2.98	3.15
E : A	3.09	3.35
DTE (m/s)	5.72	6.30
DTE slope (m/s^2^)	9.88	10.01
E′ (m/s)	4.87	6.53
A′ (m/s)	3.61	3.09
AV max (m/s)	1.45	1.76
PV max (m/s)	1.23	1.57
GLPS_LAX (%)	9.32	10.45
GLPS_4CH (%)	8.21	9.04
GLPS_2CH (%)	11.23	12.12
GLPS_AVE (%)	9.76	10.54
SC (%)	3.98	6.09
SrCs (/s)	5.98	6.84
SrCE (/s)	5.42	6.01
SR (%)	4.97	8.45
SrRs (/s)	5.89	9.10
SrRE (/s)	8.84	10.68
LVEDV (mL)	3.56	5.69
LVESV (mL)	5.32	6.77
LV SV (mL)	5.13	5.74
LV EF (%)	4.98	5.44
LV mass (g)	3.89	4.12
LV native T1 time (ms)	2.89	3.77
LV post T1 time (ms)	1.59	1.78
LV ECV (%)	2.12	3.45

Abbreviations: A, peak velocity of late diastolic transmitral flow; A′, peak late diastolic velocity of lateral mitral annulus; Ao, aortic short‐axis dimension in diastole; AV max, peak aortic flow velocity; DTE, deceleration time of early diastolic transmitral flow; DTE slope, slope of DTE; E, peak velocity of early diastolic transmitral flow; E′, peak early diastolic velocity of lateral mitral annulus; E : A, ratio between E and A; GLPS_AVE, overall average of global longitudinal strain obtained from 3 apical long‐axis views; GLPS_2CH, global longitudinal strain from apical 2‐chamber view; GLPS_4CH, global longitudinal strain from aplical 4‐chamber view; GLPS_LAX, global longitudinal strain from apical long‐axis view; IVSd, interventricular septal thickness in diastole; IVSs, interventricular septal thickness in systole; LAD_Max_, maximum left atrial septal‐to‐free wall dimension in right parasternal long‐axis 4‐chamber view; LA_Min_, minimum left atrial septal‐to‐free wall dimension in right parasternal long‐axis 4‐chamber view; LA_SAX_, left atrium short‐axis diameter in diastole; LAA emptying, peak velocity blood flow leaving the left auricular appendage during atrial contraction; LAA filling, peak velocity of blood flow entering the left auricular appendage after atrial contraction; LA : Ao, ratio between LA_SAX_ and Ao; LV ECV, left ventricular extracellular volume fraction; LVEDV, left ventricular end‐diastolic volume; LV EF, left ventricular ejection fraction; LVESV, left ventricular end‐systolic volume; LA FS, left atrial fractional shortening; LV FS, left ventricular fractional shortening; LVFWd, left ventricular free wall thickness in diastole; LVFWs, left ventricular free wall thickness in systole; LVIDd, left ventricular internal dimension in diastole; LVIDs, left ventricular internal dimension in systole; LV SV, left ventricular stroke volume; PV max, peak pulmonic flow velocity; SrCE, peak circumferential strain rate during early diastole; SrRE, peak radial strain rate during early diastole.

Within the healthy control group, no diastolic variables were significantly correlated with age, ECV, LV mass, or native T1 time. Linear measurements of the left ventricle and left atrium in diastole were significantly correlated with mass (LVIDd; *r* = 0.63, *P* = .01, LAd_LAX; *r* = 0.54, *P* = .04) and global radial and circumferential strain were significantly correlated with ECV (SR; *r* = 0.55, *P* = .04, SC; *r* = −0.69, *P* = .005).

**FIGURE 2:**
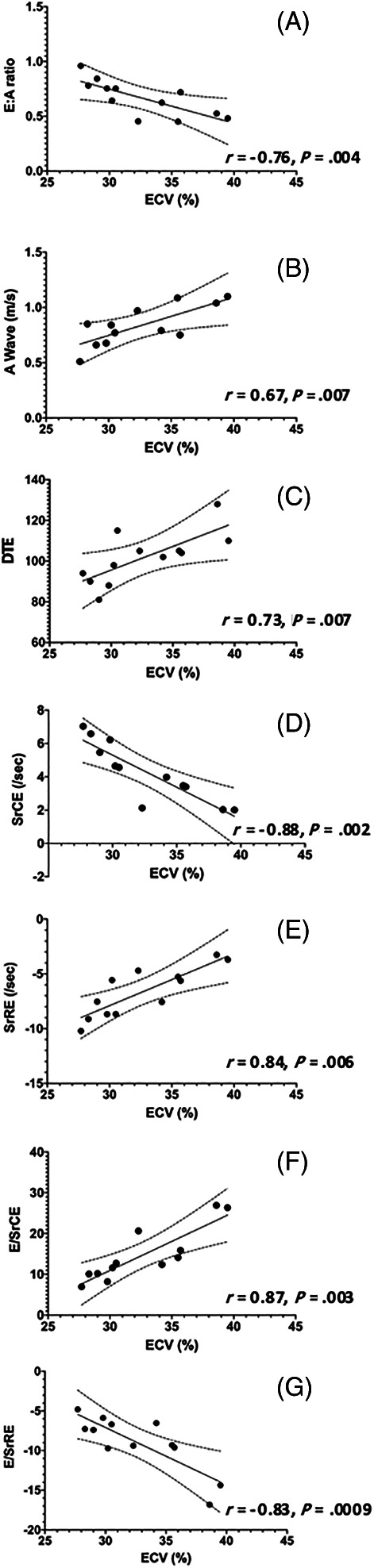
Linear regression scatter diagrams with 99% confidence interval (CI) to compared extracellular volume fraction (ECV) with (A) ratio of peak velocity of early to peak velocity late diastolic transmitral flow(E : A), (B) peak velocity of late diastolic transmitral flow (A wave), (C) deceleration time of early diastolic transmitral flow (DTE), (D) peak circumferential strain rate during early diastole (SrCE), (E) peak radial strain rate during early diastole (SrRE), (F) ratio between E and SrCE (E/SrCE), and (G) ratio between E and SrRE (E/SrRE)

**FIGURE 3:**
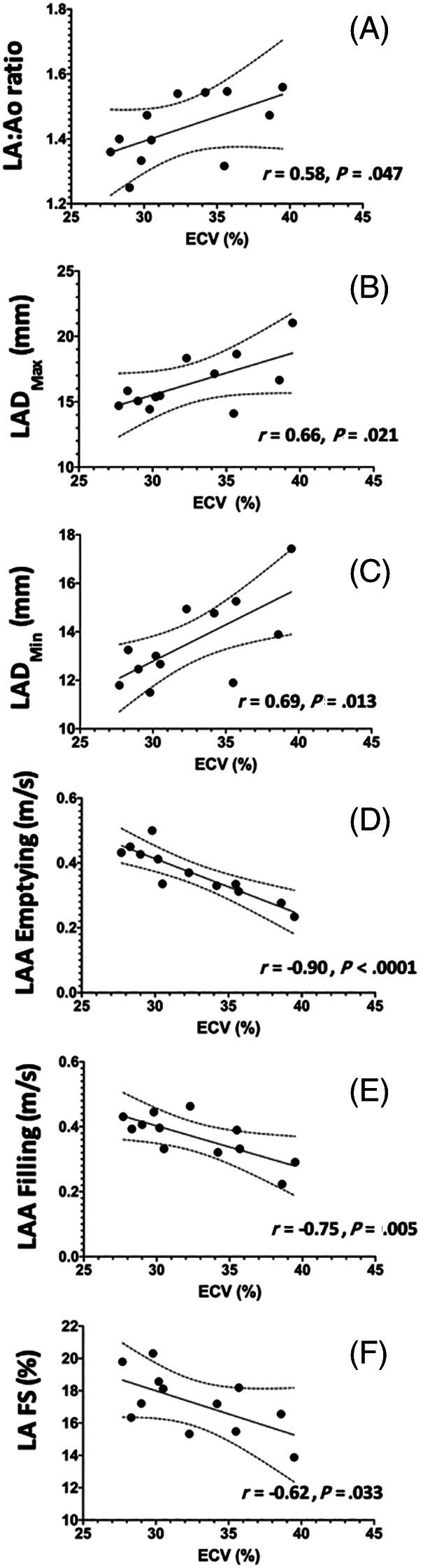
Linear regression scatter diagrams with 99% confidence interval (CI) to compared extracellular volume fraction (ECV) with (A) ratio between left atrial diameter and aorta at end diastole in the right parasternal short‐axis view (LA : Ao), (B) maximum left atrial septal‐to‐free wall dimension in right parasternal long‐axis 4‐chamber view (LAD_Max_), (C) minimum left atrial septal‐to‐free wall dimension in right parasternal long‐axis 4‐chamber view (LA_Min_), (D) peak velocity blood flow leaving the left auricular appendage during atrial contraction (LAA emptying), (E) peak velocity of blood flow entering the left auricular appendage after atrial contraction (LAA filling), and (F) left atrial fractional shortening (LA FS)

**FIGURE 4:**
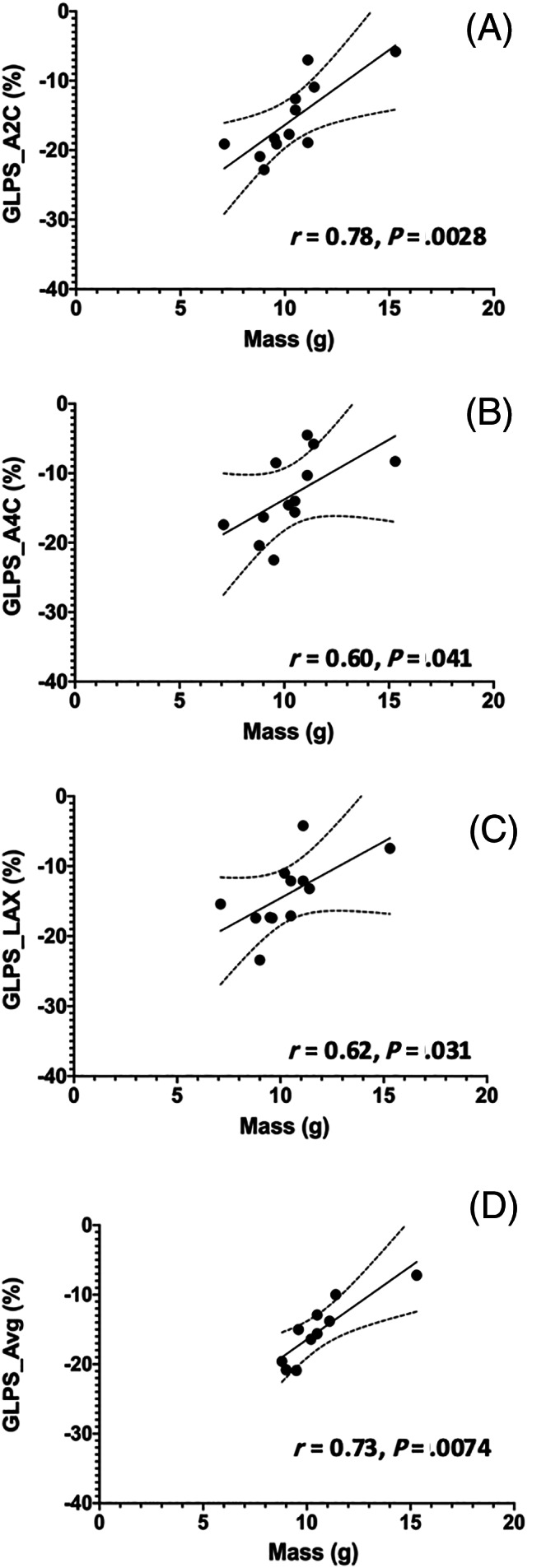
Linear regression scatter diagrams with 99% confidence interval (CI) to compared left ventricular mass with (A) global longitudinal strain from apical 2‐chamber view (GLPS_2CH), (B) global longitudinal strain from apical 4‐chamber view (GLPS_4CH), (C) global longitudinal strain from apical long‐axis view (GLPS_LAX), and (D) overall average of global longitudinal strain obtained from 3 apical long‐axis views (GLPS_AVE)

Within the HCM group, there were significant correlations with multiple echocardiographic variables for both ECV and mass summarized in Table 5. Many indices of diastolic function (DTE, A, E : A, SrCE, SrRE, E/SrCE, and E/SrRE) were correlated with ECV (Figure 2). None of these indices were significantly correlated with echocardiographic IVSd, LVFWd, or CMR derived LV mass. The results of multiple linear regression for evaluating the relationship among age, ECV, and indices of diagnostic and left atrial function indicate that ECV, but not age, is a significant factor for diastolic function in this population (Table 6). Additionally, left atrial linear dimensions (LAd_LAX, LAS_LAX, and LA : Ao) as well as left atrial function (LA_FS%_LAX, LAu Fill, and LAu Empty) were significantly correlated with ECV and no other variables (Figure 3). Indices of systolic global longitudinal strain (Figure 4) were correlated with LV mass. It should be noted that some echocardiographic values (GLPS, SC, SrRE, and E/SrRE) are negative, therefore any positive correlations with these indices indicates a negative relationship. The cats in this study were considered to have symmetrical left ventricular hypertrophy, as there was no statistical difference between septal and free wall thickness (*P* = .08).

**TABLE 5:** Pearson's correlation for selected echocardiographic and cardiac magnetic resonance imaging variable in 12 cats with hypertrophic cardiomyopathy (HCM)

CMR Variable	Echocardiographic Variable	*r*	*P*
ECV	DTE	0.73	.007
ECV	A	0.76	.004
ECV	E : A	−0.76	.004
ECV	SrCE	−0.88	.0002
ECV	SrRE	0.84	.006
ECV	E/SrCE	0.87	.006
ECV	E/SrRE	−0.83	.0009
ECV	LAD_Max_	0.66	.02
ECV	LAD_Min_	0.69	.01
ECV	LA FS	−0.62	.03
ECV	LA : Ao	0.58	.05
ECV	LAA filling	−0.75	.005
ECV	LAA emptying	−0.90	<.0001
LV mass	GLPS_AVE	0.73	.007
LV mass	GLPS_LAX	0.62	.03
LV mass	GLPS_4CH	0.60	.04
LV mass	GLPS_2CH	0.78	.002

Abbreviations: A, peak velocity of late diastolic transmitral flow; Ao, aortic short‐axis dimension in diastole; DTE, deceleration time of early diastolic transmitral flow; E, peak velocity of early diastolic transmitral flow; E : A, ratio between E and A; E/SrCE, ratio between E and SrCE; E/SrRE, ratio between E and SrRE; GLPS_AVE, overall average of global longitudinal strain obtained from 3 apical long‐axis views; GLPS_2CH, global longitudinal strain from apical 2‐chamber view; GLPS_4CH, global longitudinal strain from aplical 4‐chamber view; GLPS_LAX, global longitudinal strain from apical long‐axis view; LAD_Max_, maximum left atrial septal‐to‐free wall dimension in right parasternal long‐axis 4‐chamber view; LA_Min_, minimum left atrial septal‐to‐free wall dimension in right parasternal long‐axis 4‐chamber view; LAA emptying, peak velocity blood flow leaving the left auricular appendage during atrial contraction; LAA filling, peak velocity of blood flow entering the left auricular appendage after atrial contraction; LA : Ao, ratio between left atrium short‐axis diameter in short axis and Ao; LA FS, left atrial fractional shortening; *r*, Pearson's correlation coefficient; SrCE, peak circumferential strain rate during early diastole; SrRE, peak radial strain rate during early diastole.

**TABLE 6:** Multiple linear regression of age and ECV on selected echocardiographic variables in 12 cats with hypertrophic cardiomyopathy (HCM)

Dependent variable	Independent variables	ß	*P*
DTE	ECV	2.27	.005
	Age	−0.075	.11
A	ECV	0.035	.007
	Age	0.000019	.98
E : A	ECV	−0.031	.005
	Age	−0.00058	.44
SrCE	ECV	−0.39	.0004
	Age	−0.0022	.65
SrRE	ECV	0.48	.0009
	Age	0.0055	.44
E/SrCE	ECV	1.41	.002
	Age	−0.029	.09
E/SrRE	ECV	−0.71	.01
	Age	0.012	.26
LA FS	ECV	−0.29	.04
	Age	−0.00012	.98

Abbreviations: A, peak velocity of late diastolic transmitral flow; DTE, deceleration time of early diastolic transmitral flow; E, peak velocity of early diastolic transmitral flow; E : A, ratio between E and A; ECV, extracellular volme fraction; E/SrCE, ratio between E and SrCE; E/SrRE, ratio between E and SrRE; LA FS, left atrial fractional shortening; SrCE, peak circumferential strain rate during early diastole; SrRE, peak radial strain rate during early diastole.

Receiver operating characteristic results indicate native T1 time (AUC = 0.95, 95% CI = 0.87‐1.00, *P* < .001), ECV (AUC = 0.96, 95% CI = 0.91‐1.00, *P* < .001), and LV mass (AUC = 0.98, 95% CI = 0.94‐1.00, *P* < .001) provide outstanding discernment between healthy and HCM groups. Using a cutoff for native T1 of >1153 ms (Youden's Index = 80) provides a sensitivity of 92% and specificity 88%, whereas a cutoff for ECV of >28.1% (Youden's Index = 74) provides a sensitivity of 92% and specificity of 82%, and for LV mass a cutoff of >8.2 g (Youden's Index = 92) provides a sensitivity of 92% and specificity of 100%.

## 4 DISCUSSION

This study demonstrated that CMR measures of the cardiac fibrosis (native T1 and ECV) are obtainable in cats, with acceptable repeatability, and are significantly different between healthy and HCM cats. In this study sample, HCM cats had increased native T1 times and ECV compared with normal cats, but only ECV was significantly correlated with diastolic function and left atrial size. Preclinical HCM cats had reduced GLPS compared with normal cats and this variable was not correlated with fibrosis.

Based on CMR, HCM cats in this study sample have increased interstitial fibrosis compared with normal cats. Native T1 and ECV are highly correlated with histological measures of collagen and fibrosis in humans with a variety of cardiac diseases including HCM.^17^, ^24^, ^25^, ^26^ Native T1 is a composite signal of both the cellular and extracellular components of the myocardium, whereas ECV is a more specific assessment of the extracellular matrix alone and therefore a measure of interstitial fibrosis. Therefore, native T1 does not differentiate cellular hypertrophy from diffuse fibrosis whereas ECV does. Histopathology was not performed in this study as all animals are currently alive at the time of writing. Therefore, although we cannot be sure what is causing expansion of the interstitial matrix, historical postmortem studies indicate interstitial fibrosis, rather than amyloidosis, myocarditis, or other etiologies, is a relatively common finding in cats with HCM.^4^, ^27^ Additionally, follow‐up evaluations for all the HCM affected cats demonstrates persistent left ventricular hypertrophy in all cats, ruling out transient myocardial thickening and other causes of myocardial edema.

The role of CMR in veterinary medicine is limited, but provides novel information about cardiac composition that cannot be determined from echocardiography. Although none of the cats in this study were in heart failure, there is reason to believe that diffuse fibrosis plays an important role in disease progression.^27^ In people with heart failure and preserved ejection fraction, higher ECV is associated with a higher rate of all‐cause mortality and first heart failure hospitalization.^28^ Further studies are warranted to determine if ECV can aid in the detection of HCM in cats, in particular for cats with secondary left ventricular hypertrophy and equivocal echocardiographic findings, and if ECV confers prognostic information.

Results of this study also demonstrated that ECV was correlated with multiple measures of diastolic function, LA size, and LA function irrespective of age, LV mass, IVSd, or LVFWd. Native T1 did not correlate with diastolic function and is likely related to the composite signal (cellular and extracellular) that determines native T1 time. The relationship between ECV and diastolic dysfunction occurs in people with clinical heart failure and asymptomatic HCM.^29^, ^30^, ^31^ Additionally, in humans abnormal ECV values have been correlated with increased ventricular filling pressure measured noninvasively with echocardiography in HCM, suggesting that diffuse fibrosis plays an important role in the pathophysiology of diastolic dysfunction.^29^, ^32^ Furthermore, in humans with heart failure with preserved systolic function, there is a correlation between the amount of collagen type 1 found on endomyocardial biopsy and echocardiographic indices of diastolic dysfunction.^33^ Utilizing DTE, A, E : A, SrCE, SrRE, E/SrCE, and E/SrRE, our study suggests a mechanistic link between higher ECV in HCM cats and impaired diastolic function. This relationship was independent of LV mass and echocardiographic derived wall thickness, illustrating the importance of fibrosis on diastolic function and the inability of linear wall thickness measurements from echocardiography to predict diffuse fibrosis. The preclinical HCM cats in this study were at most moderately affected and it remains to be seen if this relationship would be true in more severely affected cats. Interestingly, LA function was correlated with ECV and not measures of wall thickness or mass, suggesting that expansion of the interstitium and ultimately distensibility may play a role in LA size and function. The relationship between LV ECV and impaired LA function is also present in humans with non‐ischemic cardiomyopathy, chronic obstructive pulmonary disease, and systemic hypertension.^34^, ^35^, ^36^

Similar to other veterinary studies, despite clinically normal measures of global systolic function (LV FS), indices of systolic function measured by strain (SC, SR, and GLPS) support an overall decreased systolic function in cats with HCM.^37^, ^38^ Impairment of contractile function in HCM assessed by GLPS was associated with the LV mass, but not markers of extracellular fibrosis. These results suggest that impairment of contractility in HCM is mediated by mechanisms other than extracellular expansion and is consistent with results of regional strain imaging in people with HCM.^39^ In humans, increased ECV correlates with impaired diastolic function in patients with HCM but not systolic heart failure, suggesting a more predominant role of interstitial fibrosis in diastolic dysfunction.^40^

Our study had several limitations. This study included a small number of cats and further studies are warranted to determine the repeatability of these results. Additionally, the sample size of this study does not allow for the establishment of reference ranges and importantly, native T1 and ECV values should only be compared if they are obtained under similar conditions, including acquisition scheme, field strength, and processing approach. Future studies are needed to establish reference ranges for normal cats at the most common MRI field strengths 1.5T and 3T. We used echocardiography as the reference standard to determine if a cat was affected; however, it is still possible that some healthy cats are affected but have not progressed. Additionally, the preclinical HCM cats in this study were at most moderately affected and findings could differ in cats with more advanced HCM. In particularly, the relatively mild nature of the preclinical HCM could influence the distribution of fibrosis as well as the relationship between diastolic function and LV wall thickness. There were no significant differences with respect to the distribution of fibrosis based on CMR. On possible explanation for this finding could be related to the severity of HCM. Cats in this study were at most moderately affected and fibrosis could be more diffuse in the earlier stages of HCM. Additionally, the HCM affected cats in this study were considered to have symmetrical left ventricular hypertrophy, not allowing for comparison of regionally thicker areas of myocardium. The ROC results in this study are for detecting HCM based on the echocardiographic criteria, and it remains to be seen if CMR is more sensitive than echocardiography at discriminating between affected and unaffected cats in an equivocal range. Histopathology was not performed in this study, therefore even though ECV has been correlated with histopathology in multiple other species, we cannot definitely conclude that ECV has the same relationship in cats. Finally, correlations in this study do not equate to outcomes and prospective studies are warranted to determine the outcome relationship between ECV and HCM.

In this study sample, cats with HCM had significantly higher ECV values compared with healthy controls and ECV significantly correlated with worsening diastolic function and left atrial size. Quantitative assessment of ECV is feasible in cats and can provide additional information not available using standard imaging techniques. Larger prospective studies are necessary to validate our findings and the effect of ECV on prognosis in cats with HCM requires further study.

## CONFLICT OF INTEREST DECLARATION

Authors declare no conflict of interest.

## OFF‐LABEL ANTIMICROBIAL DECLARATION

Authors declare no off‐label use of antimicrobials.

## INSTITUTIONAL ANIMAL CARE AND USE COMMITTEE (IACUC) OR OTHER APPROVAL DECLARATION

Approved by the IACUC of the University of Illinois at Urban‐Champaign, protocol #17281.

## HUMAN ETHICS APPROVAL DECLARATION

Authors declare human ethics approval was not needed for this study.

## References

[1] Paige CF , Abbott JA , Elvinger F , Pyle RL . Prevalence of cardiomyopathy in apparently healthy cats. J Am Vet Med Assoc. 2009;234:1398‐1403. 19480619 10.2460/javma.234.11.1398

[2] Payne JR , Borgeat K , Connolly DJ , et al. Prognostic indicators in cats with hypertrophic cardiomyopathy. J Vet Intern Med. 2013;27:1427‐1436. 24134821 10.1111/jvim.12215

[3] Fox PR , Liu SK , Maron BJ . Echocardiographic assessment of spontaneously occurring feline hypertrophic cardiomyopathy. An animal model of human disease. Circulation. 1995;92:2645‐2651. 7586368 10.1161/01.cir.92.9.2645

[4] Liu SK , Roberts WC , Maron BJ . Comparison of morphologic findings in spontaneously occurring hypertrophic cardiomyopathy in humans, cats and dogs. Am J Cardiol. 1993;72:944‐951. 8213553 10.1016/0002-9149(93)91112-u

[5] Martos R , Baugh J , Ledwidge M , et al. Diastolic heart failure: evidence of increased myocardial collagen turnover linked to diastolic dysfunction. Circulation. 2007;115:888‐895. 17283265 10.1161/CIRCULATIONAHA.106.638569

[6] Weber KT , Brilla CG . Pathological hypertrophy and cardiac interstitium. Fibrosis and renin‐angiotensin‐aldosterone system. Circulation. 1991;83:1849‐1865. 1828192 10.1161/01.cir.83.6.1849

[7] Adabag AS , Maron BJ , Appelbaum E , et al. Occurrence and frequency of arrhythmias in hypertrophic cardiomyopathy in relation to delayed enhancement on cardiovascular magnetic resonance. J Am Coll Cardiol. 2008;51:1369‐1374. 18387438 10.1016/j.jacc.2007.11.071

[8] Hamlin SA , Henry TS , Little BP , Lerakis S , Stillman AE . Mapping the future of cardiac MR imaging: case‐based review of T1 and T2 mapping techniques. Radiographics. 2014;34:1594‐1611. 25310419 10.1148/rg.346140030

[9] Choudhury L , Mahrholdt H , Wagner A , et al. Myocardial scarring in asymptomatic or mildly symptomatic patients with hypertrophic cardiomyopathy. J Am Coll Cardiol. 2002;40:2156‐2164. 12505229 10.1016/s0735-1097(02)02602-5

[10] Moon JCC , Reed E , Sheppard MN , et al. The histologic basis of late gadolinium enhancement cardiovascular magnetic resonance in hypertrophic cardiomyopathy. J Am Coll Cardiol. 2004;43:2260‐2264. 15193690 10.1016/j.jacc.2004.03.035

[11] Wilson JM , Villareal RP , Hariharan R , Massumi A , Muthupillai R , Flamm SD . Magnetic resonance imaging of myocardial fibrosis in hypertrophic cardiomyopathy. Tex Heart Inst J. 2002;29:176‐180. 12224720 PMC124756

[12] MacDonald KA , Wisner ER , Larson RF , et al. Comparison of myocardial contrast enhancement via cardiac magnetic resonance imaging in healthy cats and cats with hypertrophic cardiomyopathy. Am J Vet Res. 2005;66:1891‐1894. 16334945 10.2460/ajvr.2005.66.1891

[13] Haaf P , Garg P , Messroghli DR , et al. Cardiac T1 mapping and extracellular volume (ECV) in clinical practice: a comprehensive review. J Cardiovasc Magn Reson. 2016;18:89. 27899132 10.1186/s12968-016-0308-4 PMC5129251

[14] Parsai C , O'Hanlon R , Prasad SK , Mohiaddin RH . Diagnostic and prognostic value of cardiovascular magnetic resonance in non‐ischaemic cardiomyopathies. J Cardiovasc Magn Reson. 2012;14:54. 22857649 10.1186/1532-429X-14-54 PMC3436728

[15] Taylor AJ , Salerno M , Dharmakumar R , Jerosch‐Herold M . T1 mapping: basic techniques and clinical applications. JACC Cardiovasc Imaging. 2016;9:67‐81. 26762877 10.1016/j.jcmg.2015.11.005

[16] Sibley CT , Noureldin RA , Gai N , et al. T1 mapping in cardiomyopathy at cardiac MR: comparison with endomyocardial biopsy. Radiology. 2012;265:724‐732. 23091172 10.1148/radiol.12112721 PMC3504318

[17] Kammerlander AA , Marzluf BA , Zotter‐Tufaro C , et al. T1 mapping by CMR imaging: from histological validation to clinical implication. JACC Cardiovasc Imaging. 2016;9:14‐23. 26684970 10.1016/j.jcmg.2015.11.002

[18] Zhou Z , Xu L , Wang R , et al. Quantification of doxorubicin‐induced interstitial myocardial fibrosis in a beagle model using equilibrium contrast‐enhanced computed tomography: a comparative study with cardiac magnetic resonance T1‐mapping. Int J Cardiol. 2019;281:150‐155. 30738608 10.1016/j.ijcard.2019.01.021

[19] Dass S , Suttie JJ , Piechnik SK , et al. Myocardial tissue characterization using magnetic resonance noncontrast t1 mapping in hypertrophic and dilated cardiomyopathy. Circ Cardiovasc Imaging. 2012;5:726‐733. 23071146 10.1161/CIRCIMAGING.112.976738

[20] Puntmann VO , Voigt T , Chen Z , et al. Native T1 mapping in differentiation of normal myocardium from diffuse disease in hypertrophic and dilated cardiomyopathy. JACC Cardiovasc Imaging. 2013;6:475‐484. 23498674 10.1016/j.jcmg.2012.08.019

[21] Häggström J , Luis Fuentes V , Wess G . Screening for hypertrophic cardiomyopathy in cats. J Vet Cardiol. 2015;17(Suppl 1):S134‐S149. 26776573 10.1016/j.jvc.2015.07.003

[22] Thomas WP , Gaber CE , Jacobs GJ , et al. Recommendations for standards in transthoracic two‐dimensional echocardiography in the dog and cat. Echocardiography Committee of the Specialty of Cardiology, American College of Veterinary Internal Medicine. J Vet Intern Med. 1993;7:247‐252. 8246215 10.1111/j.1939-1676.1993.tb01015.x

[23] Wansapura J , Fleck R , Crotty E , Gottliebson W . Frequency scouting for cardiac imaging with SSFP at 3 tesla. Pediatr Radiol. 2006;36:1082‐1085. 16830122 10.1007/s00247-006-0255-6

[24] van Heeswijk RB , Bastiaansen JAM , Iglesias JF , et al. Quantification of myocardial interstitial fibrosis and extracellular volume for the detection of cardiac allograft vasculopathy. Int J Cardiovasc Imaging. 2019;26:533‐542. 10.1007/s10554-019-01733-3 31724114

[25] de Meester de Ravenstein C , Bouzin C , Lazam S , et al. Histological validation of measurement of diffuse interstitial myocardial fibrosis by myocardial extravascular volume fraction from Modified Look‐Locker imaging (MOLLI) T1 mapping at 3 T. J Cardiovasc Magn Reson. 2015;17:48. 26062931 10.1186/s12968-015-0150-0 PMC4464705

[26] Flett AS , Hayward MP , Ashworth MT , et al. Equilibrium contrast cardiovascular magnetic resonance for the measurement of diffuse fibrosis: preliminary validation in humans. Circulation. 2010;122:138‐144. 20585010 10.1161/CIRCULATIONAHA.109.930636

[27] Fox PR . Hypertrophic cardiomyopathy. Clinical and pathologic correlates. J Vet Cardiol. 2003;5:39‐45. 19081364 10.1016/S1760-2734(06)70051-0

[28] Roy C , Slimani A , de Meester C , et al. Associations and prognostic significance of diffuse myocardial fibrosis by cardiovascular magnetic resonance in heart failure with preserved ejection fraction. J Cardiovasc Magn Reson. 2018;20:55. 30086783 10.1186/s12968-018-0477-4 PMC6081897

[29] Ellims AH , Iles LM , Ling L , Hare JL , Kaye DM , Taylor AJ . Diffuse myocardial fibrosis in hypertrophic cardiomyopathy can be identified by cardiovascular magnetic resonance, and is associated with left ventricular diastolic dysfunction. J Cardiovasc Magn Reson. 2012;14:76. 23107451 10.1186/1532-429X-14-76 PMC3502601

[30] Iles L , Pfluger H , Phrommintikul A , et al. Evaluation of diffuse myocardial fibrosis in heart failure with cardiac magnetic resonance contrast‐enhanced T1 mapping. J Am Coll Cardiol. 2008;52:1574‐1580. 19007595 10.1016/j.jacc.2008.06.049

[31] Nucifora G , Muser D , Gianfagna P , Morocutti G , Proclemer A . Systolic and diastolic myocardial mechanics in hypertrophic cardiomyopathy and their link to the extent of hypertrophy, replacement fibrosis and interstitial fibrosis. Int J Cardiovasc Imaging. 2015;31:1603‐1610. 26210792 10.1007/s10554-015-0720-0

[32] Ho CY , Abbasi SA , Neilan TG , et al. T1 measurements identify extracellular volume expansion in hypertrophic cardiomyopathy sarcomere mutation carriers with and without left ventricular hypertrophy. Circ Cardiovasc Imaging. 2013;6:415‐422. 23549607 10.1161/CIRCIMAGING.112.000333 PMC3769196

[33] Kasner M , Westermann D , Lopez B , et al. Diastolic tissue Doppler indexes correlate with the degree of collagen expression and cross‐linking in heart failure and normal ejection fraction. J Am Coll Cardiol. 2011;57:977‐985. 21329845 10.1016/j.jacc.2010.10.024

[34] Shah RV , Kato S , Roujol S , et al. Native myocardial T1 as a biomarker of cardiac structure in non‐ischemic cardiomyopathy. Am J Cardiol. 2016;117:282‐288. 26684511 10.1016/j.amjcard.2015.10.046

[35] Rodrigues JCL , Erdei T , Dastidar AG , et al. Left ventricular extracellular volume fraction and atrioventricular interaction in hypertension. Eur Radiol. 2019;29:1574‐1585. 30232515 10.1007/s00330-018-5700-z

[36] Neilan TG , Bakker JP , Sharma B , et al. T1 measurements for detection of expansion of the myocardial extracellular volume in chronic obstructive pulmonary disease. Can J Cardiol. 2014;30:1668‐1675. 25442461 10.1016/j.cjca.2014.08.006 PMC4258158

[37] Takano H , Isogai T , Aoki T , et al. Feasibility of radial and circumferential strain analysis using 2D speckle tracking echocardiography in cats. J Vet Med Sci. 2015;77:193‐201. 25373881 10.1292/jvms.13-0241 PMC4363022

[38] Wess G , Sarkar R , Hartmann K . Assessment of left ventricular systolic function by strain imaging echocardiography in various stages of feline hypertrophic cardiomyopathy. J Vet Intern Med. 2010;24:1375‐1382. 20738767 10.1111/j.1939-1676.2010.0586.x

[39] Swoboda PP , McDiarmid AK , Erhayiem B , et al. Effect of cellular and extracellular pathology assessed by T1 mapping on regional contractile function in hypertrophic cardiomyopathy. J Cardiovasc Magn Reson. 2017;19:16. 28215181 10.1186/s12968-017-0334-x PMC5317053

[40] Su M‐YM , Lin L‐Y , Tseng Y‐HE , et al. CMR‐verified diffuse myocardial fibrosis is associated with diastolic dysfunction in HFpEF. JACC Cardiovasc Imaging. 2014;7:991‐997. 25240451 10.1016/j.jcmg.2014.04.022

